# Chylothorax after Primary Repair of Esophageal Atresia with Tracheo-esophageal Fistula: Successful Management by Biological Fibrin Glue

**Published:** 2012-09-01

**Authors:** Anjan kumar Dhua, Simmi K Ratan, Satish K Aggarwal

**Affiliations:** Department of Pediatric surgery, Maulana Azad Medical College, Delhi 110002.

**Keywords:** Esophageal atresia, Tracheo-esophageal fistula, Chylothorax, Fibrin glue

## Abstract

A neonate, who had undergone primary repair of esophageal atresia with tracheo-esophageal fistula, developed right pleural effusion in the postoperative period. It was initially misdiagnosed as an anastomotic leak, but later confirmed to be chylothorax. Conservative treatment failed. Application of biological fibrin glue (sealant) on the mediastinum through a thoracotomy was curative.

## INTRODUCTION

Chylothorax, as a complication of surgery for esophageal atresia is rare. We report a newborn who developed right sided chylothorax after repair of an esophageal atresia (EA) with tracheo-esophageal fistula (TEF). 

## CASE REPORT

A preterm (34 weeks) female baby underwent primary repair of EA with TEF in another hospital. The operation was reportedly uneventful. An intercostal chest drain and a trans-anastomotic tube were placed. On the 8th day patient developed respiratory distress with decreased air entry in the right hemithorax. Chest radiograph showed radiopaque shadow in the right hemithorax suggestive of effusion (Fig. 1a). Chest tube revealed milky fluid.

**Figure:**
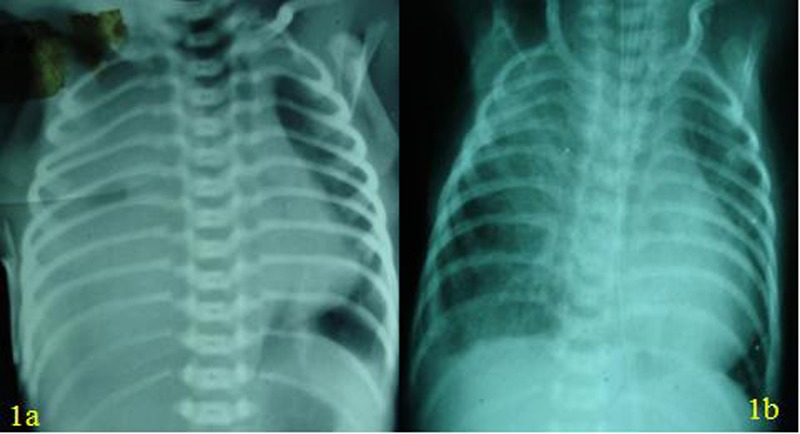
Figure: (1a) Radiograph showing radio-opaque shadow in right hemithorax with obliteration of cardio-phrenic and costo-phrenic angles. (1b) Post-operative radiograph showing significant improvement of right lung zones

A diagnosis of anastomotic leak was made and conservative management was followed for the next 17 days. Feeds were continued through the trans-anastomotic tube, but the chest tube continued to drain fluid. On the 26th day a re-exploration was planned because of failed conservative treatment. However, on parents request the baby was referred to us for further management.

Patient was a baby girl with a weight of 1030 grams. She was hemodynamically stable. A chest tube was reinserted which was removed earlier. It drained serous fluid. Blood investigations showed Hemoglobin of 13 gm%, total white cell counts 15000/mm3. A sepsis screen was positive. Gavage feeding was started. On the following day the output from the chest tube increased and became cloudy. Biochemical analysis of the fluid showed 661.5 mg/dl of triglycerides against a serum triglyceride level of 104.5 mg/dl. A diagnosis of chylothorax was made. The baby had already received ceftriaxone and meropenem. Meropenem was continued. Blood culture did not grow any organism. For the next four days the chest drain produced more than 70 ml fluid per day. The baby was in a catabolic phase. Total parenteral nutrition (TPN) was not considered useful as the patient had already spent almost four weeks in the hospitals and was on antibiotics for nearly the entire period. Septic complications of TPN were thought to be too risky. It was decided to operate upon the patient. 

Right postero-lateral thoracotomy was performed on the 7th day of admission. A trans-pleural approach was used. The anastomosis was intact. Clear lymphatic fluid found leaking from the mediastinum diffusely below the anastomosis. No clear leak point could be identified. Two ml of fibrin sealant was applied over the area. The clot covered the entire mediastinal surface and the lymphatic leak stopped. A chest tube of size 10Fr was left and the thorax closed. Post operatively the baby was electively ventilated for three days. The chest drain produced 10 ml serous fluid in the first 24 hours but remained dry subsequently.

Gavage feeding was started on the fifth post operative day and gradually increased to bring the baby on full enteral feeds on the 10th day. The chest tube was then removed. Chest radiograph showed both lungs inflated [Fig. 1b]. Weight gain was documented. However, the baby became oxygen dependent. She needed ventilator support to maintain saturation. A flexible bronchoscopy revealed tracheo-bronchomalcia. An aortopexy was planned but the child developed pneumonitis and septicemia and expired 45 days after second surgery.

## DISCUSSION

Chylothorax is the accumulation of lymphatic fluid in the pleural space that usually occurs after injury to the thoracic duct (surgery or trauma) or in association with various medical conditions including neoplasm, lymphatic or a congenital abnormality [1]. 

Conservative treatment includes keeping the child starved, or giving enteral medium chain triglycerides with or without intravenous alimentation. This treatment had failed in our baby. Initial misdiagnosis (as a leak) resulted in a delay of about 2 weeks in instituting aggressive conservative management of chylothorax. During this period the baby had lost weight. Components of usual surgical treatment are: treatment of the underlying cause, decreasing chyle production, draining and obliterating the pleural space, suture closure of thoracic duct or application of sealing agents, providing appropriate fluid and nutritional replacement, and instituting necessary respiratory care.

Surgical therapy is reserved for cases with persistent and/or high volume lymph leak that does not resolve within 4 weeks of conservative management. Various surgical procedures have been described including direct ligation of the thoracic duct [2, 3] pleurodesis with different agents including application of fibrin glue to putative sites of leaks [4] and placement of a pleuro-peritoneal shunt [5, 6]. Congenital chylothorax has been reported to be successfully treated by the application of fibrin glue [7]. 

Among all surgical options, application of fibrin glue appears to be an attractive approach in high risk cases like neonates. It is quick, safe, effective and easy for the novice. Its efficacy has been shown in postoperative chylothorax cases. Nguyen and Tchervenkov in 1994 have reported successful application of fibrin glue in a 600 gram premature neonate [8]. There are few reports in English literature on the use of fibrin glue for chylothorax in neonates as a complication of esophageal atresia repair. Rifai et al in 2003 successfully treated chylothorax in a post surgical case of esophageal atresia by a combination of argon beam coagulation of the mediastinum and fibrin glue application [9]. 

High index of suspicion is necessary for diagnosing chylothorax in postoperative effusions developing after thoracic surgeries. The diagnosis may be confused with anastomotic leak in EA/TEF. Prompt treatment should be initiated with conservative methods however application of topical fibrin glue seems a safe and easy approach.

## Footnotes

**Source of Support:** Nil

**Conflict of Interest:** None declared

## References

[1] ( 2005). Doerr CH, Allen MS, Nichols FC III, Ryu JH. Etiology of chylothorax in 203 patients. Mayo Clin Proc.

[2] ( 1981). Patterson GA, Todd TR, Delarue NC, Ilves R, Pearson FG, Cooper JD. Supradiaphragmatic ligation of the thoracic duct in intractable chylous fistula. Ann Thorac Surg.

[3] ( 1983). Stenzl W, Rigler B, Tscheliessnigg KH, Beitzke A, Metzler H. Treatment of postsurgical chylothorax with fibrin glue. Thorac Cardiovasc Surg.

[4] ( 1983). Azizkhan RG, Canfield J, Alford BA, Rodgers BM. Pleuroperitoneal shunts in the management of neonatal chylothorax. J Pediatr Surg.

[5] ( 1992). Rheuban KS, Kron IL, Carpenter MA, Gutgesell HP, Rodgers BM. Pleuroperitoneal shunts for refractory chylothorax after operation for congenital heart disease. Ann Thorac Surg.

[6] ( 2009). Mathur NB, Singh B, Kumar A, Aggarwal SK. Successful Treatment of congenital chylothorax using fibrin glue. Indian J Pediatr.

[7] ( 2008). Panthongviriyakul C, Bines JE. Post-operative chylothorax in children: an evidence-based management algorithm. J Paediatr Child Health.

[8] ( 1994). Nguyen D, Tchervenkov CI. Successful management of postoperative chylothorax with fibrin glue in a premature neonate. Can J Surg.

[9] ( 2003). Rifai N, Sfeir R, Rakza T, Alameh J, Besson R, Lequien P, Storme L. Successful management of severe chylothorax with argon plasma fulguration and fibrin glue in a premature infant. Eur J Pediatr Surg.

